# Loop diuretics association with Alzheimer’s disease risk

**DOI:** 10.3389/fragi.2023.1211571

**Published:** 2023-09-25

**Authors:** Anna Graber-Naidich, Justin Lee, Kyan Younes, Michael D. Greicius, Yann Le Guen, Zihuai He

**Affiliations:** ^1^ Quantitative Sciences Unit, Department of Medicine (Biomedical Informatics Research), Stanford University, Stanford, CA, United States; ^2^ Department of Neurology and Neurological Sciences, Stanford University, Stanford, CA, United States

**Keywords:** Alzheimer’s disease, bumetanide, electronic health record informatics, furosemide, quantitative pharmacology

## Abstract

**Objectives:** To investigate whether exposure history to two common loop diuretics, bumetanide and furosemide, affects the risk of developing Alzheimer’s disease (AD) after accounting for socioeconomic status and congestive heart failure.

**Methods:** Individuals exposed to bumetanide or furosemide were identified in the Stanford University electronic health record using the de-identified Observational Medical Outcomes Partnership platform. We matched the AD case cohort to a control cohort (1:20 case:control) on gender, race, ethnicity, and hypertension, and controlled for variables that could potentially be collinear with bumetanide exposure and/or AD diagnosis. Among individuals older than 65 years, 5,839 AD cases and 116,103 matched controls were included. A total of 1,759 patients (54 cases and 1,705 controls) were exposed to bumetanide.

**Results:** After adjusting for socioeconomic status and other confounders, the exposure of bumetanide and furosemide was significantly associated with reduced AD risk (respectively, bumetanide odds ratio [OR] = 0.23; 95% confidence interval [CI], 0.15–0.36; *p* = 4.0 × 10^−11^; furosemide OR = 0.42; 95% CI, 0.38–0.47; *p* < 2.0 × 10^−16^).

**Discussion:** Our study replicates in an independent sample that a history of bumetanide exposure is associated with reduced AD risk while also highlighting an association of the most common loop diuretic (furosemide) with reduced AD risk. These associations need to be additionally replicated, and the mechanism of action remains to be investigated.

## Introduction

Medical systems generate massive amounts of electronic health record (EHR) data, and researchers have analyzed these data to derive new insights and improve healthcare (Rajkomar et al., 2019; Shah et al., 2019). Stanford University has established a novel and secure data platform: Observational Medical Outcomes Partnership (OMOP) Common Data Model (CDM). Alzheimer’s disease (AD) is well suited for analysis with OMOP given its multifaceted complexity, prevalence, and the multitude of small sample size studies that claim benefit for certain interventions.

AD is a neurodegenerative disorder of uncertain cause and pathogenesis. In the United States, as many as one in nine people (10.7%) older than 65 years has AD (Rajan et al., 2021). Recently, repurposing bumetanide as an AD medication was proposed based on data that showed bumetanide “reversed” *APOE* genotype-dependent transcriptomic signatures in mouse and cell culture models (Taubes et al., 2021). This finding was investigated in two EHR-based cohorts demonstrating that in individuals older than 65 years, bumetanide exposure was associated with lower AD prevalence (Taubes et al., 2021). This finding warrants further validation as bumetanide is more expensive than the commonly prescribed loop diuretic (furosemide), and thus, potential socioeconomic status (SES) confounding such as insurance coverage needs to be investigated. Both furosemide and bumetanide are indicated, and often interchangeably used, for patients with hypertension, congestive heart failure (CHF), and kidney disease. In this study, using Stanford’s EHR data, we sought to replicate the bumetanide findings in an independent dataset accounting for SES, hypertension, and CHF, and additionally test the association of furosemide with AD risk.

## Methods

Stanford’s de-identified OMOP instance hosts multi-factor and multi-modal data, including Stanford’s structured clinical data, clinical notes, meta-data on clinical notes, extracted concepts from clinical notes using natural language processing and other approaches, and radiological images. Participants or their caregivers provided written informed consent to store their data in OMOP. The Stanford University institutional review board granted the current study protocol an exemption because the analyses were carried out on de-identified data; therefore, additional informed consent was not required.

We have curated OMOP data for 656,683 patients older than 65 years at their last known visit. We focused on 5,872 patients with AD defined by ICD9 and ICD10 codes (ICD10: G30.1, G30.8, G30.9, and ICD9: 331.0, Table 1). We matched individual AD patients with up to 20 controls on age and exact match of sex, race, ethnicity, and hypertension (Table 2) using R package *optmatch* (Hansen and Olsen Klopfer, 2006). We excluded 937 matched controls with an age difference greater than 5 years and 373 matched controls that belonged to strata with fewer than 15 controls per case, resulting in 5,839 AD cases and 116,103 matched controls.

**TABLE 1:** ICD9 and ICD10 code description.

ICD9/10 code	Description	Number of patients with this diagnosis code as their first Alzheimer’s diagnosis	
		Male	Female
G30.1	Alzheimer disease with late onset	86	128
G30.8	Other Alzheimer’s disease	76	148
G30.9	Alzheimer’s disease, unspecified	705	1062
331	Alzheimer’s disease	804	1380
I11.0	Hypertensive heart disease with heart failure	1223	1775
I13.0	Hypertensive heart and chronic kidney disease with heart failure and stage 1 through stage 4 chronic kidney disease, or unspecified chronic kidney disease	1406	1485
I13.2	Hypertensive heart and chronic kidney disease with heart failure and with stage 5 chronic kidney disease, or end-stage renal disease	129	144

ICD9 and ICD10 codes at first diagnosis.

**TABLE 2:** Demographics of participants by diagnosis status.

	AD case	Matched control	SMD
**N**	5,839	116,103	
**Age at the last visit (median [IQR])**	84.00 [79.00, 88.00]	83.00 [79.00, 87.00]	0.058
**Gender (%)**			0.005
**Female, n (%)**	3,583 (61.4)	70,969 (61.1)	
**Male, n (%)**	2,256 (38.6)	45,134 (38.9)	
**Race (%)**			0.013
**White, n (%)**	3,636 (62.3)	72,719 (62.6)	
**Asian, n (%)**	835 (14.3)	16,633 (14.3)	
**Black or African American, n (%)**	262 (4.5)	4,961 (4.3)	
**Native Hawaiian or other Pacific Islander, n (%)**	38 (0.7)	700 (0.6)	
**American Indian or Alaska Native, n (%)**	4 (0.1)	80 (0.1)	
**Patient refused, n (%)**	65 (1.1)	1271 (1.1)	
**Other, n (%)**	755 (12.9)	14,869 (12.8)	
**Unknown, n (%)**	205 (3.5)	4,100 (3.5)	
**No matching concept, n (%)**	39 (0.7)	770 (0.7)	
**Ethnicity (%)**			0.001
**Not Hispanic or Latino, n (%)**	5,022 (86.0)	99,824 (86.0)	
**Hispanic or Latino, n (%)**	437 (7.5)	8,717 (7.5)	
**No matching concept, n (%)**	380 (6.5)	7,562 (6.5)	
**Hypertension (%)**	3,667 (62.8)	72,663 (62.6)	0.004
**Congestive heart failure (%)**	414 (7.1)	5,627 (4.8)	0.095
**Median household income (by zip code) (median [IQR])**	63088.30 [48073.11, 76242.65]	55136.25 [40246.02, 74796.61]	0.219
**Ever Medicare/Medicaid/VA insurance**	5,415 (98.7)	95,593 (97.4)	0.092
**Ever private insurance**	1,414 (25.8)	24,240 (24.7)	0.025

Each AD patient was matched with up to 20 controls on age and exact match of sex, race, ethnicity, and hypertension.

## Statistical analysis

We scanned the data using the medication orders or medical history variable tables to identify those who had been exposed to bumetanide or, respectively, furosemide prior to AD diagnosis. We included any type of exposure to the drug, specifically oral or IV exposures, for any duration of time. We calculated the percent of AD cases and non-AD controls exposed to bumetanide and furosemide using a χ2 test. In addition, as *post hoc* sensitivity analyses, we calculated the odds ratio of AD diagnosis for those exposed to bumetanide (and separately furosemide) while adjusting for variables that could potentially be collinear with bumetanide exposure and/or AD diagnosis including diagnosis of CHF (defined by the ICD10 of I11, I13, and I50, Table 1), insurance type, and median income (defined by the patient’s recorded zip code, derived from publicly available data from the United States Census Bureau), and explored the relationship of AD with the other commonly used loop diuretic, furosemide. Statistical analyses were performed using R (version 3.6.3). The results are shown in Tables 3A,B.

**TABLE 3:** (AB) Bumetanide and furosemide exposure details of exposed participants by diagnosis status (A). Bumetanide exposures for full matched cohort (B) and furosemide exposures for full matched cohort.

Characteristic	Case, N = 27	Control, N = 1,705
Duration of bumetanide exposure (days), median (IQR)	115 (6, 682)	21 (2, 334)
Days from first bumetanide exposure to first AD diagnosis, median (IQR)	225 (1, 978)	NA (NA, NA)
Ever exposed to bumetanide via oral route, n (%)	25 (93)	1,405 (82)
Ever exposed to bumetanide via oral route, n (%)	11 (41)	776 (46)
Ever exposed to 0.25 mg/mL dosage injectable solution, n (%)	11 (41)	768 (45)
Ever exposed to 0.5 mg dosage oral tablet, n (%)	10 (37)	302 (18)
Ever exposed to 1 mg dosage oral tablet, n (%)	22 (81)	1,079 (63)
Ever exposed to 2 mg dosage oral tablet, n (%)	11 (41)	587 (34)

Characteristic	Case, N = 633	Control, N = 20,017
Duration of furosemide exposure (days), median (IQR)	94 (2, 909)	25 (1, 664)
Days from first furosemide exposure to first AD diagnosis, median (IQR)	351 (12, 1,153)	NA (NA, NA)
Ever exposed to furosemide via oral route, n (%)	471 (74)	14,718 (74)
Ever exposed to furosemide via IV route, n (%)	394 (62)	12,433 (62)
Ever exposed to 8 mg/ml dosage injectable or oral solution, n (%)	5 (0.8)	128 (0.6)
Ever exposed to 10 mg/ml dosage injectable or oral solution, n (%)	394 (62)	12,449 (62)
Ever exposed to 20 mg dosage oral tablet, n (%)	397 (63)	11,786 (59)
Ever exposed to 40 mg dosage oral tablet, n (%)	204 (32)	6,775 (34)
Ever exposed to 80 mg dosage oral tablet, n (%)	26 (4.1)	980 (4.9)

## Results

A total of 1,732 patients (27 cases and 1,705 controls) were exposed to bumetanide during any of their visits (prior to their AD diagnosis for cases). For the full-matched cohort, among AD cases, 0.5% (27/5839) of patients were exposed to bumetanide prior to diagnosis compared to 1.5% (1705/116103) of controls exposed to bumetanide, suggesting patients exposed to bumetanide are less likely to develop AD. The unadjusted odds ratio (OR) for AD diagnosis among bumetanide exposed was 0.31 (95% CI, 0.21–0.46; *p* = 2.4 × 10^−9^).

To adjust SES variables, we included insurance information (if patients ever had Medicare/Medicaid/VA and if patients ever had private insurance) and median household income by the zip code. However, we did have some missingness in our data—insurance information was available for 94.0% of cases and 84.5% of controls. In addition, for the zip code data informing the median income component, we focused on patients from California, and due to de-identification reasons, data were only available for 76.8% of cases and 74.1% of controls. Because SES estimates were not easily imputable from our data, a sensitivity analysis was performed as a complete case analysis. In this sensitivity analysis, we restricted the cohort to patients with complete insurance and median income data (N = 77,688) and repeated the unadjusted analysis prior to fitting a multivariable model adjusted for CHF, insurance, and median income. In the complete case-restricted cohort, 21/4238 (0.50%) AD cases and 1,321/73,450 (1.8%) matched controls were exposed to bumetanide. The unadjusted OR remained similar to that in our primary analysis (OR = 0.27; 95% CI, 0.18–0.42; *p* = 3.7 × 10^−9^). After adjusting for CHF, insurance, and median income the estimated OR for AD diagnosis among bumetanide exposed was 0.23 (95% CI, 0.15–0.36; *p* = 4.0 × 10^−11^).

For the full-matched cohort, among AD cases, 10.8% (633/5839) of patients were exposed to furosemide prior to diagnosis compared to 17.2% (20023/116103) of controls exposed to furosemide, suggesting patients exposed to bumetanide are less likely to develop AD. The unadjusted OR for AD diagnosis among furosemide exposed was 0.58 (95% CI, 0.54–0.63; *p* < 2.0 × 10^−16^). In the same sensitivity analysis performed for bumetanide exposure, the unadjusted OR for the complete case-restricted cohort remained similar to that in our primary analysis (OR = 0.53; 95% CI, 0.48–0.58; *p* < 2.0 × 10^−16^); this protective effect was replicated after adjusting for CHF, insurance, and median income (OR = 0.42; 95% CI, 0.38–0.47; *p* < 2.0 × 10^−16^).

## Discussion

Most clinical trials in AD suffer from an inherent shortfall regarding primary prevention as they do not give insights on whether a compound reduces the incidence of AD as medications are tested after disease onset. Studying EHR using OMOP allows us to derive insight into possible primary prevention of AD (Datta et al., 2020).

In an independent dataset, our results replicate those of the original study that found a protective effect of bumetanide exposure on AD risk (Taubes et al., 2021). We further investigated whether this effect is generalizable to the more commonly used and less expensive medication in the same class and adjusted for potentially confounding variables such as SES and CHF. In our study, we calculated the odds ratio of AD diagnosis for those exposed to bumetanide (and separately furosemide) and found that the exposure of both bumetanide and furosemide was associated with reduced future AD diagnosis.

Both medications have similar indications and mechanisms of action: potential protective molecular modulation of neuronal transmembrane chloride gradients by blocking NKCC1 in the central nervous system (Kharod et al., 2019), which is the mechanism that led to proposed investigations to treat autism (Lemonnier et al., 2012), schizophrenia (Rahmanzadeh et al., 2017), and epilepsy (Eftekhari et al., 2013; Rahmanzadeh et al., 2017). Both bumetanide and furosemide have been shown to penetrate the blood–brain barrier, albeit at low concentrations (Javaheri et al., 1994; Töllner et al., 2015). The brain bumetanide concentrations following systemic administration are below those required for effective NKCC1 inhibition (Johanson et al., 1992; Holtkamp et al., 2003; Römermann et al., 2017; Brandt et al., 2018). However, other potential explanations for the protective effects including unique effects on the *APOE* genotype-dependent transcriptomic signature (Taubes et al., 2021), potent diuretic effects, and off-target metabolic, cardiorespiratory, and hormonal alterations that may be indirectly linked to reducing the risk of AD are also a possibility (Brater, 1991; Puskarjov et al., 2014). Our OMOP EHR dataset analysis demonstrated a potential inverse association between past bumetanide and furosemide exposures (Table 4) and AD onset. These associations remained significant even after correcting for SES and CHF, indicating that the results are not driven by differences in SES or severity of cardiac disease.

**TABLE 4:** Odds ratio for AD diagnosis among bumetanide- and furosemide-exposed participants.

Bumetanide analyses	Or (95% CI)	*p*-value
Unadjusted matched cohort bumetanide exposure prior to AD diagnosis	0.31 (0.21, 0.46)	2.4 × 10^−9^
CHF-adjusted matched cohort bumetanide exposure prior to AD diagnosis	0.25 (0.17, 0.37)	2.59 × 10^−12^
Unadjusted complete case cohort bumetanide exposure prior to AD diagnosis	0.27 (0.18, 0.42)	3.72 × 10^−9^
CHF-adjusted complete case cohort bumetanide exposure prior to AD diagnosis	0.23 (0.15, 0.35)	2.11 × 10^−11^
CHF- and SES-adjusted complete case cohort bumetanide exposure prior to AD diagnosis	0.23 (0.15, 0.36)	4.0 × 10^−11^
**Furosemide analyses**		
Unadjusted matched cohort furosemide exposure prior to AD diagnosis	0.58 (0.54, 0.63)	<2.0 × 10^−16^
CHF-adjusted matched cohort furosemide exposure prior to AD diagnosis	0.48 (0.44, 0.52)	<2.0 × 10^−16^
Unadjusted complete case cohort furosemide exposure prior to AD diagnosis	0.53 (0.48, 0.58)	<2.0 × 10^−16^
CHF-adjusted complete case cohort furosemide exposure prior to AD diagnosis	0.43 (0.39, 0.48)	<2.0 × 10^−16^
CHF- and SES-adjusted complete case cohort furosemide exposure prior to AD diagnosis	0.42 (0.38, 0.47)	<2.0 × 10^−16^

Individuals were older than 65 years, and the odds ratio was adjusted for age, sex, race, ethnicity, hypertension, and with and without adjusting CHF and SES (insurance and median income).

AD, Alzheimer’s disease; CHF, congestive heart failure; SES, socioeconomic status; OR, odds ratio.

These results should be treated cautiously as they are based on retrospective data. Bumetanide and furosemide are potent loop diuretics that if given excessively, can lead to a profound diuresis with water and electrolyte depletion, which is particularly problematic in the elderly population. In addition, insurance and income were modeled through proxies available in OMOP and may not fully account for differences in SES. Last, additional functional studies are warranted to investigate the biological mechanism through which bumetanide and furosemide exposures are associated with reduced AD risk. The current findings do not support the use of bumetanide for the prevention or treatment of AD. There is a need for prospective, randomized, double-blinded, and placebo-controlled clinical trials to confirm the findings in patients without comorbidities and determine the lowest effective dose that may reduce the risk of AD without causing intolerable side effects.

## Data Availability

The data analyzed in this study are subject to the following licenses/restrictions: Electronic Health Record from Stanford University. Requests to access these datasets should be directed to zihuai@stanford.edu.
